# A Severe Case of Disseminated Multifocal Methicillin-Susceptible Staphylococcal Infection in a Diabetic Patient

**DOI:** 10.7759/cureus.23483

**Published:** 2022-03-25

**Authors:** Mohamed H Fayed, Haris Iftikhar, Shahzad Anjum, Omar Khyatt, Mavia Najam

**Affiliations:** 1 Emergency Medicine, Hamad Medical Corporation, Doha, QAT; 2 Radiology, Hamad Medical Corporation, Doha, QAT; 3 Medical Education, Hamad Medical Corporation, Doha, QAT

**Keywords:** septic emboli, metastatic infection, bacteremia, mrsa, mssa, staphylococcus aureus, disseminated

## Abstract

Methicillin-susceptible *Staphylococcus aureus *(MSSA) is quite common in the environment. It can lead to a wide range of infections varying from simple boils to disseminated and metastatic infections. Disseminated multifocal MSSA infection without infective endocarditis is extremely rare. We report a case of a 48-year-old diabetic male who presented with complaints of back pain, lower limb weakness, urinary retention, and saddle sensation loss. His imaging showed an epidural abscess, spondylitis, multiple paraspinous abscess collections, iliopsoas and gluteal abscess formation, multiple abdominal abscesses, multiple cavitating lung nodules, left-sided empyema, and azygos venous thrombosis. He was managed with urgent laminectomy and evacuation of spinal epidural abscess. He was admitted under the medical team for further multidisciplinary patient care. Emergency physicians and internists should be able to recognize such cases early on to make appropriate management plans. Misdiagnosis and delay in treatment initiation can lead to high mortality and poor patient outcomes. Advanced imaging techniques should be utilized to avoid missed foci. Improved source control results in better patient outcomes.

## Introduction

*Staphylococcus aureus* is well known to cause a wide variety of infections. It can range from minor infections like a boil to disseminated infections involving multiple organs. The notorious adaptive ability of *Staphylococcus aureus* led to the emergence of methicillin-resistant *Staphylococcus aureus *(MRSA) in the early 1960s [1]. Although physicians are concerned about the possibility of MRSA, they should not forget that the methicillin-susceptible *Staphylococcus aureus *(MSSA) is equally virulent and can lead to disseminated infections. MSSA is quite common, with 20-30% of the population is being a carrier of this bacterium. It is present in the nasopharynx, skin, gastrointestinal tract, and perineum [2]. Risk factors common for both MSSA and MRSA bacteremia include immunosuppression, organ transplantation, cancer, dialysis dependence, and diabetes mellitus [3].

Disseminated multifocal MSSA infection without infective endocarditis is extremely rare. One study with a primary diagnosis of *Staphylococcus aureus *bacteremia (SAB) in 1440 patients reported only one case of disseminated MSSA infection without infective endocarditis [3]. We want to report a case of a 48-year-old male who presented with a severe disseminated MSSA infection in our emergency department. Emergency physicians and internists should be able to recognize such cases early on to make appropriate treatment and disposition plans. These rare cases typically need a multidisciplinary approach for management and source control. If unrecognized or not adequately treated, these patients can have a high mortality rate and poor outcomes.

## Case presentation

**Figure 1:**
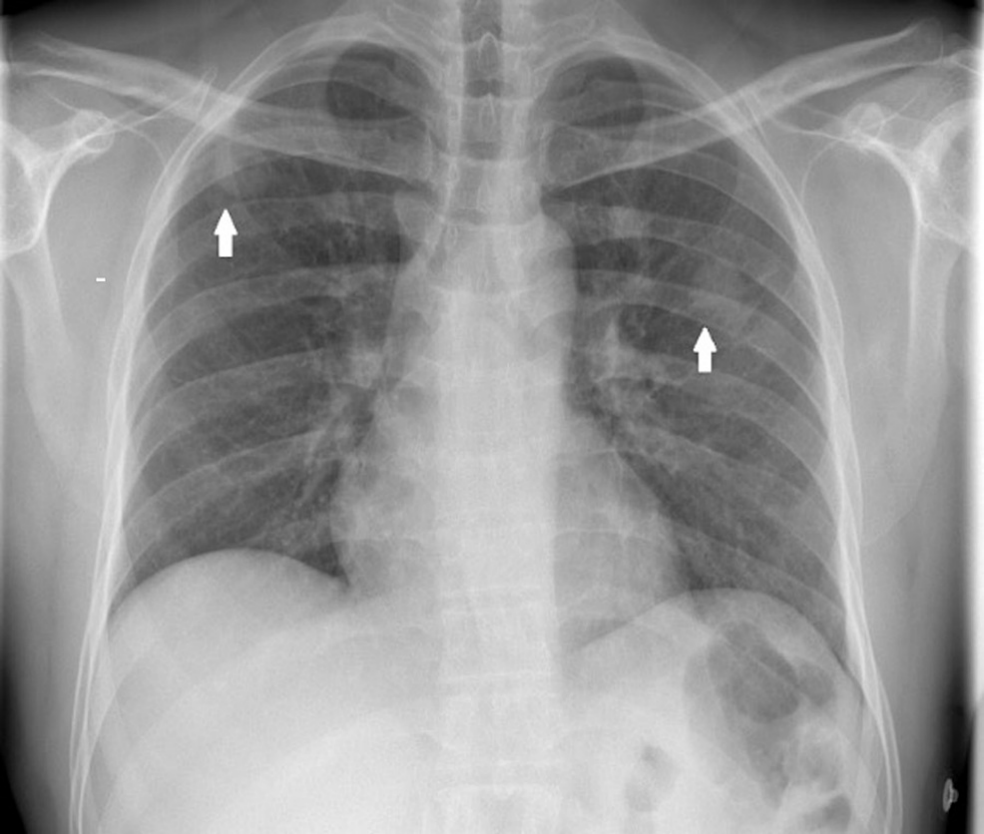
Posteroanterior view of chest radiograph. The image shows faint two rounded nodular opacities in the right upper and left middle zones (arrows). There is subtle lucency in the right upper nodule likely representing breakdown (small cavity).

A 48-year-old male known case with type 2 diabetes mellitus, on oral hypoglycemic, and a chronic smoker presented with complaints of back pain and lower limb weakness for one day. He was also having urinary retention and subjective saddle sensory loss. He had back pain for a few weeks with recent worsening. He visited the emergency department twice last month for chest pain, investigated, and was found to have two faint lung nodules (Figure 1). He was referred to pulmonology for urgent screening for cancer. He reported a weight loss of 5 kg in the last two months. He denied fever, night sweats, cough, dyspnea, dizziness, syncope, or convulsions. He denied any allergies.

On physical examination, he had a temperature of 37.1°C, tachycardia at 136 beats per minute, a respiratory rate of 25 breaths per minute, oxygen saturation of 99% on room air, and blood pressure of 125/90 mmHg. He was conscious, alert, and oriented to person, place, and time with a Glasgow Coma Scale (GCS) score of 15. His sensations were decreased in both lower limbs at level L1 and below. His digital rectal examination showed decreased tone and sensation. His motor strength as per the Medical Research Council’s classification system was normal in both upper limbs and decreased in lower limbs (Table 1).

**Table 1:** Power of different muscle groups of lower limbs.

Lower limb muscle group	Right	Left
Hip flexor	2	3
Hip extension	3	4
Knee flex	2	3
Knee extensor	3	3
Ankle dorsiflexor	4	4
Ankle plantar flexor	4	4
Extensor hallucis longus	4	4

His abdominal examination showed a distended bladder. Point-of-care ultrasound showed bilateral mild hydronephrosis with distended bladder and confirmed urinary retention. The rest of his physical examination was unremarkable.

An urgent neurosurgical consult was made with a working diagnosis of spinal abscess or metastatic disease. He received analgesics, intravenous fluids, broad-spectrum antibiotics, and high-dose dexamethasone (16 mg). A foley catheter was inserted that drained 1500 mL of urine. His initial labs showed a picture of sepsis with very high inflammatory markers (white blood cells: 28.9 × 10^9^/L, absolute neutrophil count: 25.7 × 10^9^/L, C-reactive protein: 391 mg/L), low sodium (121 mmol/L), high potassium (5.7 mmol/L), high blood glucose (16.6 mmol/L), normal pH, and mildly deranged liver function tests. Urgent MRI thoracic, lumbar and sacral spine with contrast was requested by neurosurgery.

**Figure 2:**
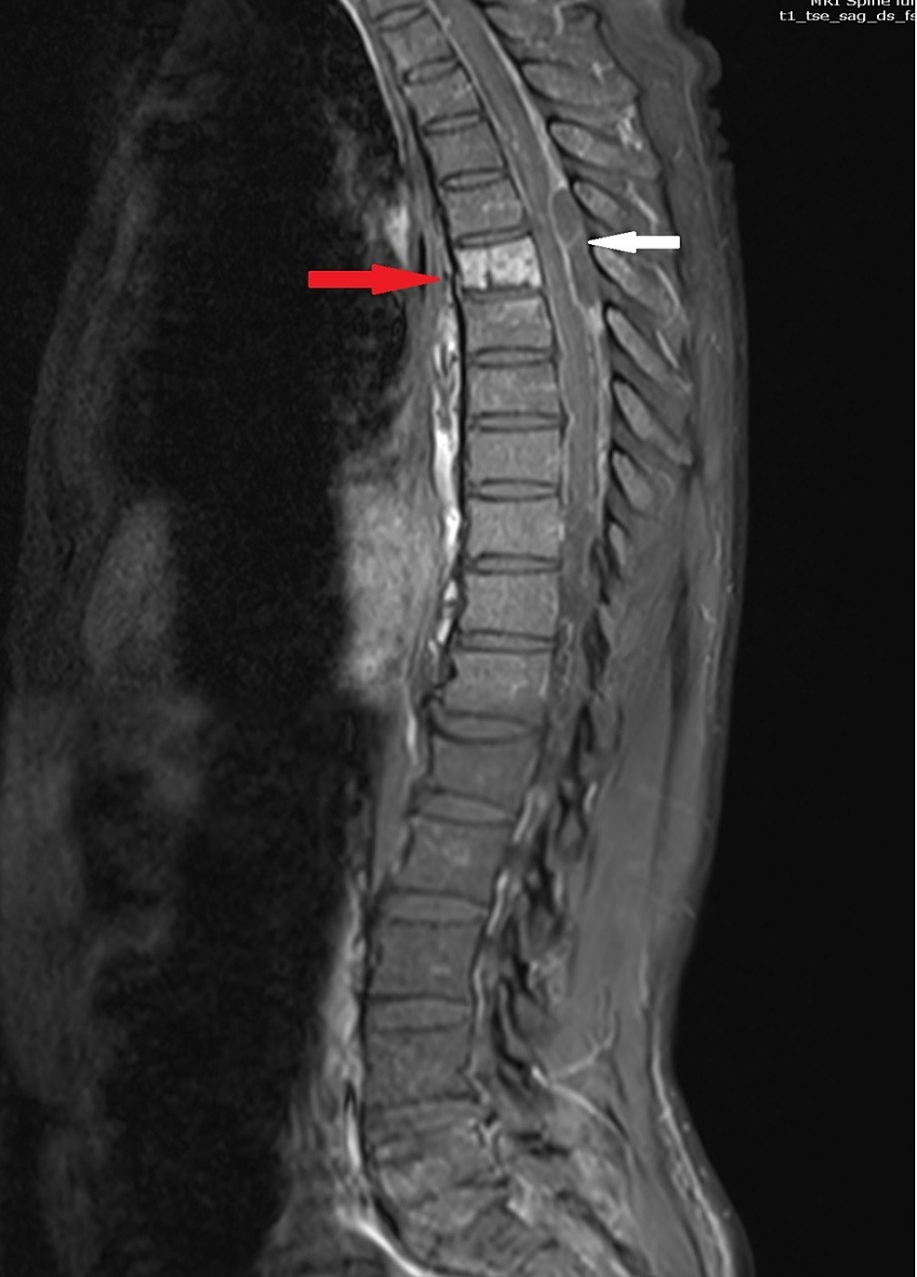
Magnetic resonance imaging, sagittal view, of dorsal and lumbar spine (post-gadolinium). The image shows peripheral enhancing collection in epidural space (epidural abscess, white arrow) indenting dorsal cord. Heterogenous enhancement of vertebral body (red arrow) suggesting spondylitis.

**Figure 3:**
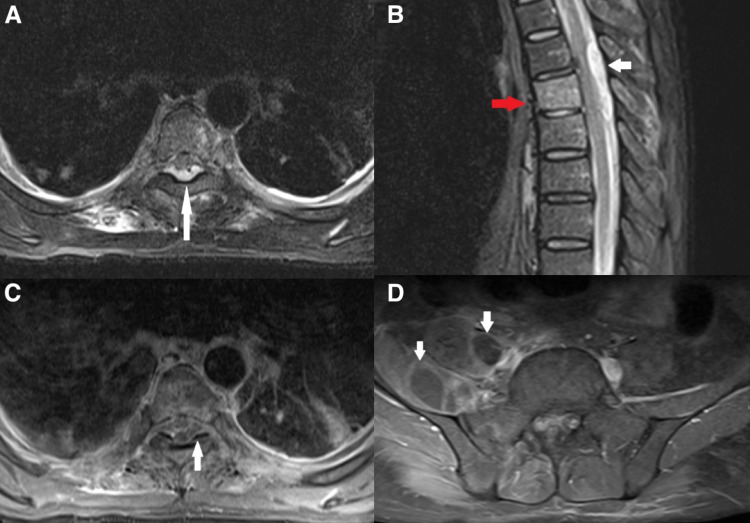
Magnetic resonance imaging of dorsal spinal cord. (A) MRI dorsal spine T2-weighted image (axial section) shows there is high signal intensity fluid in posterior epidural space with mass effect on the spinal cord (white arrow). (B) Sagittal dorsal spine (short tau inversion recovery {STIR} sequence) shows posterior epidural collection indenting posterior aspect cord (white arrow). Abnormally increased signal intensity in dorsal vertebral body (red arrow) suggesting spondylitis. (C) MRI axial section (post-gadolinium) shows peripheral enhancing collection in epidural space (white arrow: abscess) indenting compressing posterior aspect cord. (D) MRI axial section (post-gadolinium) shows peripherally enhancing collection in the right psoas and iliacus muscle (white arrows).

MRI showed an epidural abscess at the level of T5-T7 dorsal vertebra with the compressive effect on the dorsal spinal cord (Figures 2, 3A-3D). Increased contrast enhancement was noted at the epidural space between the cervical and lower thoracic level representing the extension of the inflammatory changes. Spondylitis at T6 vertebra with paraspinous soft tissue involvement at this level. MRI also showed multiple paraspinous abscess collections most prominent at the right iliopsoas muscle, the right deepest part of the psoas muscle. Between the L1 and S1 vertebral level deepest part of the psoas muscle, right erector spina muscle, and quadratus lumborum muscles have an increased post-contrast enhancement representing inflammatory signal changes together with abscess formation at the right iliopsoas muscles and prominent multiloculated collections the largest pocket measuring approximately 5 x 3 cm. Another abscess formation was noted in the left gluteal muscle. There were multiple cavitating lung nodules that may suggest septic emboli or pulmonary abscesses. There was also evidence of left-sided empyema.

**Figure 4:**
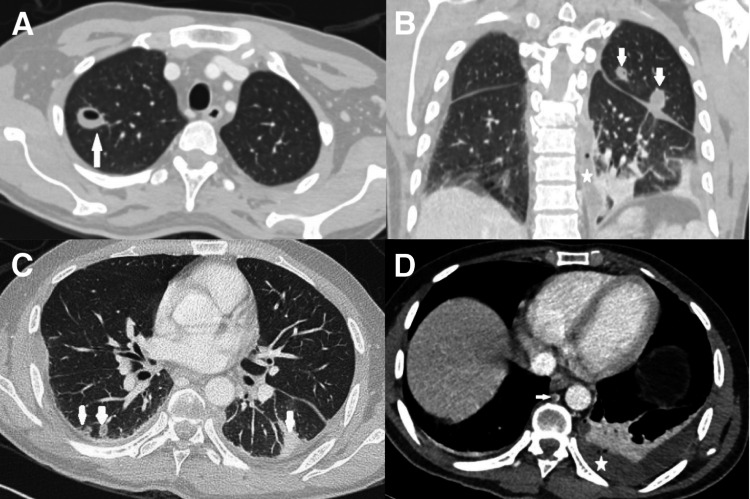
CT chest, axial and coronal sections, with contrast of the patient. CT scan chest (A) axial section (lung window) shows a small cavitary lesion in the apical segment of right upper lobe (arrow) and (B) coronal section shows two nodules in the left lung (arrows). There is a left para-vertebral collection (asterisk). CT chest (C) axial section (lung window) shows bilateral lower lobes small nodules (arrows) and necrotic changes seen within the right lower nodules, and (D) mediastinal window shows a left pleural collection with enhancing pleura keeping with empyema (asterisk). There is a filling defect within the azygos vein (thrombus shown by arrow).

**Figure 5:**
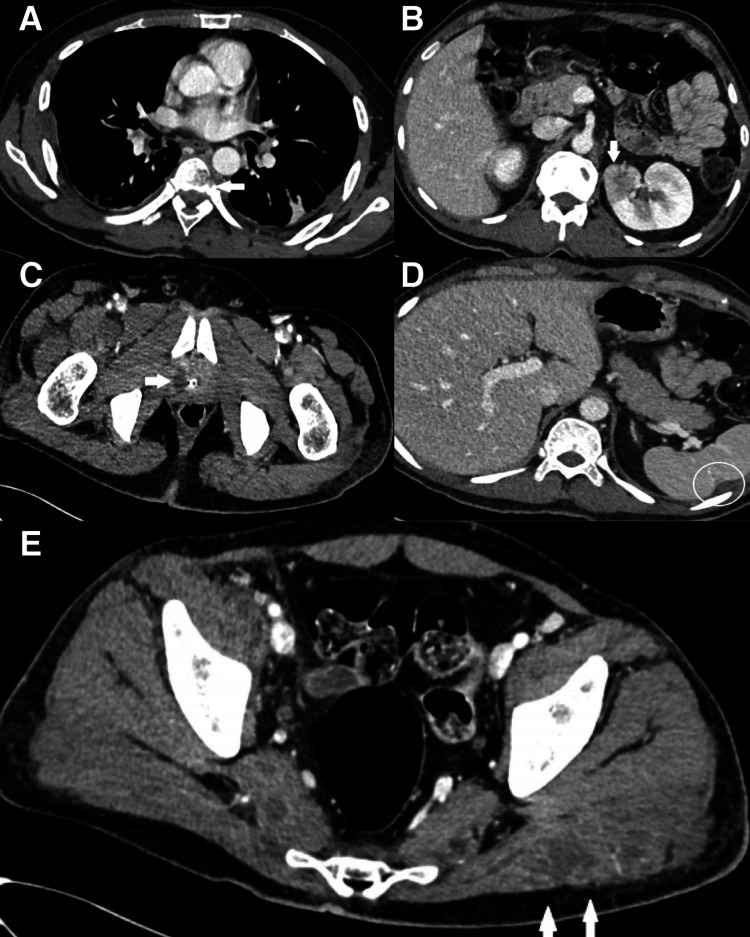
CT chest, abdomen, and pelvis (axial section) with contrast of the patient. (A) CT chest axial section shows a left paravertebral collection with evidence of vertebral bone destruction (arrow). (B) CT abdomen axial section (portal phase) shows a hypodensity in the upper pole of the left kidney with evidence of tiny low attenuation foci suggesting microabscesses (arrow). (C) CT pelvis axial section at the level of prostate shows a small fluid pocket with peripheral enhancement right lobe of the prostate (arrow) representing a small prostatic abscess. (D) CT abdomen axial section (portal phase) shows a tiny hypodense area mid spleen with a trace of adjacent fluid suggesting a small abscess (circle). (E) CT pelvis axial section shows evidence of a left gluteal abscess (arrows) and another abscess is seen in the right psoas muscle.

Following MRI spine findings, CT thorax, abdomen, and pelvis with contrast were done which showed multiple cavitating lung nodules, left-sided empyema, and azygos venous thrombosis (Figures 4A-4D, 5A-5E). Patchy hypodensities were seen in both kidneys and ill-defined hypodensity at the upper pole of the left kidney, concerning for early forming abscess. Multiple small collections, some are multiloculated, are seen scattered throughout the abdomen and pelvis involving the spleen, the prostate, the right iliopsoas muscle, and both gluteal muscles.

**Figure 6:**
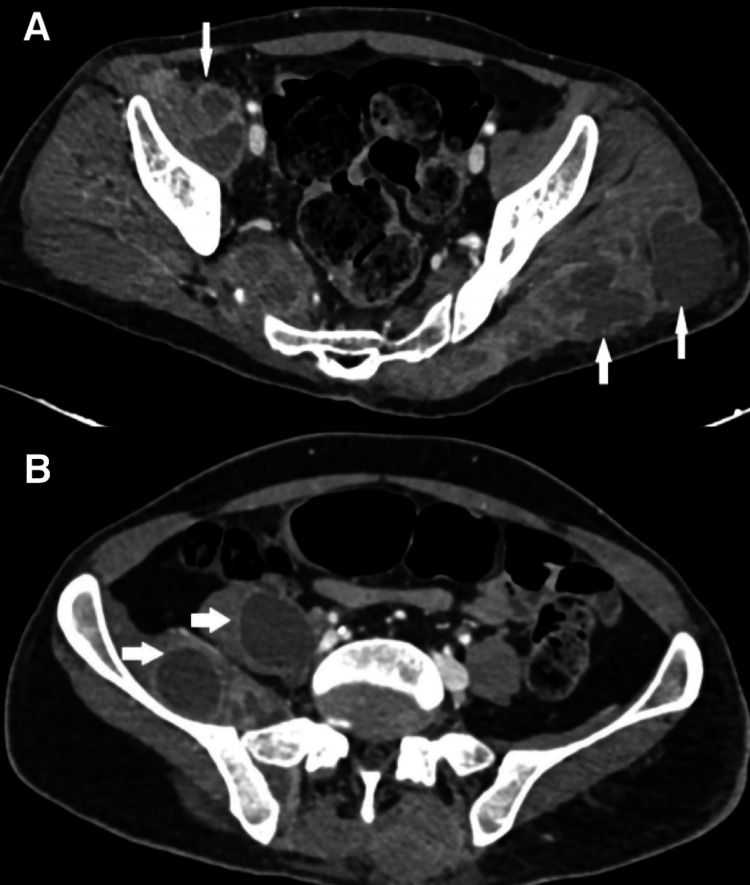
Follow-up CT abdomen and pelvis, axial section, of the patient. The image shows (A) an interval increase in the left gluteal abscess and right iliac and right obturator abscesses (arrows) and (B) an interval increase in the right psoas and iliac abscess (arrows).

The patient underwent urgent T6 laminectomy and evacuation of spinal epidural abscess. He was admitted under the medical team for further multidisciplinary patient care. His blood and abscess culture showed Staphylococcus aureus. His HbA1c was 8.6 and his HIV antigen/antibody test was non-reactive. He was initially continued on vancomycin which was later changed to intravenous cloxacillin on the finding of MSSA (methicillin-susceptible *Staphylococcus aureus*). He was started on a therapeutic dose of enoxaparin for splenic infarct and azygos vein thrombosis. Transthoracic and transesophageal echo was done which ruled out infective endocarditis. Infectious disease (ID) and thoracic surgery team advised image-guided aspiration for left pleural collection/empyema. An ultrasound (US)-guided pigtail catheter was inserted by interventional radiology for drainage. His CT abdomen was repeated which showed an interval increase in the size of the diffuse scattered loculated abscesses and a significant interval increase in the left gluteal abscess (Figures 6A, 6B). He underwent US-guided drainage of the left gluteal abscess with pigtail catheter insertion.

After a prolonged inpatient course of one month, the patient was shifted to a rehabilitation center for further care. His neurological assessment showed that the patient was continent for bladder and bowel function. His motor power in right lower extremity showed 3/5 in the hip, 2/5 in the knee, and 3/5 in the ankle. His left lower extremity power was 3/5 in the hip, 3/5 in the knee, and 3/5 in the ankle. He was able to walk with a walking frame for 60 meters under close supervision. He was not stable to walk alone. He remained admitted for three weeks in rehabilitation. On discharge, he has good muscle power (5/5) in his bilateral upper extremities, gross and fine motor skills were intact with no complaints. He was ambulating with a steady gait using a walker. He was also able to ambulate slowly without a walker. His motor power in right lower extremity showed 4/5 in the hip, 5/5 in the knee, and 5/5 in the ankle. His left lower extremity power was 5/5 in the hip, 5/5 in the knee, and 5/5 in the ankle. He lost follow-up after discharge and probably went back to his home country.

## Discussion

*Staphylococcus aureus *is one of the major causes of bacteremia. It is often associated with high morbidity and mortality. Our case report describes a case that has an initial atypical presentation of well-circumscribed lung nodules that raised the suspicion for possible malignancy. The patient later presented with spinal epidural abscess and multiple cavitating lung nodules, left-sided empyema, venous thrombosis, and multiple other deep-seated abscesses in the body. He had a disseminated disease with multiple metastatic *Staphylococcus aureus* infections which is very rare.

Metastatic *Staphylococcus aureus *infection is “a deep, distal, or secondary infection, anatomically unrelated to the primary site infection”. This includes infective endocarditis, septic arthritis, muscle abscess, epidural abscess, pyogenic spondylitis, septic pulmonary abscess, etc. [4]. The incidence of metastatic infection caused by *Staphylococcus aureus *ranges from 13% to 39% but disseminated metastatic infections are extremely rare. Two retrospective studies determined the predictive factors for metastatic infections. These include persistent fever for more than 72 hours, community-onset of infection, delay in treatment, and high C-reactive protein (CRP) levels for more than two weeks after the beginning of antibiotics [5-6].

*Staphylococcus aureus* may produce several types of molecules that are responsible for its pathogenesis and virulence. The suggested mechanism of *Staphylococcus aureus *disseminated infection and abscess formation involves the secretion of coagulases and von Willebrand factor binding protein (vWbp). They promote coagulation and change normal hemostasis thus altering host defenses. These proteins activate the hemostatic factor prothrombin and divert its activation away from host control. The bacterial surface displays agglutinins that bind fibrinogen to convert it into fibrin by proteolysis. High levels of vWbp and coagulase accumulate at the peripheries of the abscess. These molecules help in the formation of a pseudocapsule or fibrous capsule to create a mechanical barrier. This barrier prevents phagocytosis of bacteria by host neutrophils and other immune cells. There are other secreted factors that cause the recruitment and destruction of immune cells. This will transform abscesses into purulent exudate, with which *Staphylococcus aureus *disseminate to produce new infectious lesions. These are the main virulence mechanisms for the pathogenesis of SAB and the formation of abscess lesions [7].

The first-line treatment for MSSA bacteremia and metastatic infections is the semi-synthetic penicillinase-stable beta-lactam antibiotics such as nafcillin and cloxacillin. Alternatives include cefazolin and vancomycin. Cefazolin has comparable efficacy to nafcillin but vancomycin is less effective and should be reserved for MRSA. It should be de-escalated as soon as sensitivities are available. Vancomycin is associated with delayed clearance and higher mortality in these patients as compared to the above antibiotics [2,8]. Uncomplicated *Staphylococcus aureus *bacteremia (SAB) is treated for at least 14 days. The treatment should be prolonged for four to six weeks if there is a deep-seated infection. The optimum management for metastatic infection requires adequate antibiotic therapy and, if possible, the removal or drainage of the source of infection [9].

Identification of the focus of SAB and early source control can improve outcomes in metastatic and disseminated infections. The majority of relapse of SAB is associated with a failure to adequately achieve source control. This might be limited by the ability to detect a focus of infection. Transthoracic and transesophageal echocardiogram is routinely used as a screening tool in disseminated infections to rule out infective endocarditis. Advanced imaging techniques including computed tomography (CT), fluorodeoxyglucose positron emission tomography (PET), and magnetic resonance imaging (MRI) can be utilized to avoid the missed foci. Improved source control results in better patient outcomes [10].

## Conclusions

MSSA is quite common in the environment and can lead to disseminated and metastatic infections. Septic pulmonary emboli should be kept in differentials of bilateral pulmonary nodules. Misdiagnosis and delay in treatment initiation can lead to high mortality and poor patient outcomes. Emergency physicians and internists should be able to recognize such cases early on to make multidisciplinary treatment and disposition plans. The first-line treatment option for MSSA bacteremia and metastatic infections is the penicillinase-stable beta-lactam antibiotics such as nafcillin and cloxacillin. Cefazolin and vancomycin are alternatives. Advanced imaging techniques should be utilized to avoid missed foci. Improved source control results in better patient outcomes.
